# Commentary: Key Aspects of Multimodal Prehabilitation in Surgical Patients With Cancer. A Practical Approach to Integrating Resistance Exercise Programs

**DOI:** 10.1177/01632787231218993

**Published:** 2023-11-30

**Authors:** Roberto Laza-Cagigas, Marcos Seijo, Ian Swaine, Tara Rampal, Fernando Naclerio

**Affiliations:** 1 411444 University of Greenwich, UK; 2 QuestPrehab, UK

**Keywords:** preoperative interventions, post-surgery recovery, physical activity, perception of effort

## Abstract

Surgical prehabilitation aims to optimise patients’ physiological reserves to better withstand the stress of surgery, reduce the risk of postoperative complications, and promote a faster and optimal recovery. The purpose of this commentary is to outline the key aspects of prehabilitation before surgery for cancer which seem to impact its effectiveness and wider implementation. Particular attention is paid to the role and integration of resistance training programmes as a key component of multimodal prehabilitation for patients with cancer. We firstly analyse some of the barriers currently hindering the implementation of prehabilitation programmes in the National Health Service (United Kingdom). Later, we describe essential aspects of resistance training design, such as exercise modality and order execution, volume and intensity, rest periods between sets or exercises, and workout frequency. Furthermore, we propose a methodology to use the perception of effort to control patients’ progression during a prehabilitation programme.

Surgical prehabilitation refers to preoperative interventions aimed at increasing patients’ physiological reserve so they can better withstand the stress of surgery, avoid postoperative complications, and accomplish a faster and optimal post-surgery recovery (Milder et al., 2018; Valkenet et al., 2011). Although the present manuscript will cover prehabilitation in patients with cancer awaiting surgery, it is worth noticing that prehabilitation is currently implemented in different clinical environments other than the presurgical context. Prehabilitation could, therefore, be considered an intervention aimed at optimising an individual’s health prior to facing a major stressor (e.g., chemotherapy). Accordingly, different from unimodal prehabilitation that focuses only on one aspect (e.g., medical treatment), a multimodal prehabilitation programme encompasses two or more components, including physical activity, nutrition, anxiety management, pharmacology, smoking cessation, and alcohol moderation. This article identifies and discusses gaps in the current guidelines when integrating a physical activity programme, particularly resistance exercise, for patients with cancer during preoperative periods. The purpose of this article is twofold: to address the *potential reasons justifying why surgical cancer prehabilitation is not currently embedded into the National Health Service* and to describe and offer a practical approach to integrating key aspects of resistance training design to facilitate its control and progression as an essential component of multimodal prehabilitation in patients with cancer.

## Potential Barriers to the Implementation of Multimodal Prehabilitation into the National Health Service

Multimodal prehabilitation has been recognised as an effective intervention for patients with cancer. Despite this acknowledgement and support provided by the MacMillan guidelines “Prehabilitation for people with cancer” (MacMillan, 2019)*,* prehabilitation is not yet established as a standard of care in the National Health Service in the United Kingdom. Moderate evidence supports that multimodal prehabilitation improves functional recovery in patients with cancer (McIsaac et al., 2022). Although the concept of *multimodal prehabilitation* is shared amongst healthcare professionals, there can be substantial heterogeneity amongst programmes depending on how the different components are implemented. Moreover, to add further complexity to the extrapolation of the outcomes, factors other than those inherent to the prehabilitation programme should be considered. These factors include but are not limited to the type of cancer, the patient’s comorbidities, the type of surgery, surgeon expertise, or the in-hospital post-surgical care. The listed aspects may explain why it is difficult to conclude if multimodal prehabilitation leads to improved clinical outcomes and, hence, precludes its definitive integration as part of the standard of care for patients with cancer awaiting surgery.

The available evidence (Waterland et al., 2021; Wu et al., 2021) supports the benefits prehabilitation provides to the functional capacity and quality of life of patients with cancer. However, the effects on postoperative outcomes, such as length of hospital stays and potential complications are unclear (McIsaac et al., 2022; Waterland et al., 2021). Indeed, a cancer diagnosis or the period before a major surgery may be a circumstance under which patients could be more receptive to adopting lifestyle changes and, therefore, improving their chronic conditions and quality of life.

Patients approaching major cancer surgery face challenges, including those derived from exacerbated unhealthy behaviours that contribute to carcinogenesis (such as physical inactivity, poor diet, smoking, and excessive alcohol consumption). Neoadjuvant chemoradiotherapy specifically reduces physical fitness, potentially transitioning a patient from lower to higher risk (Jack et al., 2014) while also increasing the likelihood of developing sarcopenia (Sell et al., 2020), which may ultimately result in decreased overall survival (Okuno et al., 2019).

Although preoperative interventions are conducted over a relatively short timeframe ( ∼ 4–6 weeks), significant improvements in fitness and mental health can still be achieved (Durrand et al., 2019). Appropriate exercise interventions aimed at increasing cardiorespiratory capacity and muscle strength, and improving body composition (i.e., increasing lean mass and decreasing fat mass) should be integrated with the other aspects of the multimodal approach to optimise postoperative outcomes and favour quality of life in patients with cancer (Durrand et al., 2019; Moran et al., 2016). In fact, the surgical stress response elevates tissue and organ demand for oxygen, with ‘unfit’ patients struggling to meet these requirements, placing them at increased risk for adverse outcomes (Durrand et al., 2019). Thus, the physical component of a preoperative intervention emerges as a key factor to reduce the incidence of adverse effects and optimise patients’ recovery. Designing physical training programmes for patients requires professional expertise along with careful control and adjustments based on repeated objective fitness assessments. Particularly, programmes including intermittent high-intensity exercises (the so-called ‘high-intensity interval training,’ commonly abbreviated as ‘HIIT’) performed with different exercise modalities (e.g., cycling or running), inspiratory muscle exercises or resistance training using bands or small free weights may confer advantages including greater time-efficient improvements in aerobic capacity and muscular function (Weston et al., 2016).

## Resistance Exercise-Induced Benefits in Patients with Cancer

In their recent systematic review and meta-analysis, Michael et al. (2021) reported that exercise-based prehabilitation for patients with cancer before surgery was 87.7% acceptable and 89.7% feasible, with no significant toxicities. Furthermore, the existing literature supports the notion that integrating regular exercise is safe and feasible for patients with cancer in different stages (Leal et al., 2021; Rodríguez-Cañamero et al., 2022; Toohey et al., 2022). Nonetheless, there seems not to be a consensus about the most effective training design, including a proper selection of exercises and adequate manipulation of all training variables such as volume, intensity, and inter-set rest periods (Champ et al., 2022). In fact, Singh et al. (2013) conducted a systematic review of presurgical exercise interventions, reporting significant heterogeneity between studies. Additionally, these authors suggested that a long waiting period from the completion of the exercise programme to the day of surgery could negate the benefits elicited by the intervention. Therefore, prehabilitation exercise programmes should extend as close as possible to the surgery date (Singh et al., 2013). In this regard, exercise programmes have traditionally focused on the measurement, quantification, and effects of endurance-type exercise, and less attention has been drawn towards resistance exercise modalities (e.g., lifting weights). Maybe the usually vague report of the implemented methodology to adjust essential variables such as the level of resistance (light, moderate or heavy), movement velocity, number of repetitions per set, rest periods, and the correct exercise technique, complicates the interpretation of the observed results and their association with the induced physiological adaptations in patients with cancer (Champ & Yancy, 2020). In our opinion, guidelines establishing recommendations on physical activity for patients with cancer still need further specification. In some cases, activities such as going to the “gym”, “aerobics”, and “carrying bags” without further instructions, are considered within the so-called “building strength” category (Foster et al., 2018). While recommendations for adults in the UK, from which guidelines for patients with cancer are drawn, advise performing resistance exercises “repeated to failure” (Foster et al., 2018; Foster et al., 2019).

According to the American College of Sports Medicine’s (ACSM) Complete Guide to Fitness and Health; “Resistance training (also called strength training) involves the use of a variety of activities that include free weights (barbells and dumbbells), weight machines, elastic tubing, medicine balls, stability balls, and body weight. Resistance training does not refer to one specific mode of conditioning but rather to an organized process of exercising with various types of resistance to enhance muscular fitness” (Bushman, 2017). Exercise training is considered a critical strategy to ameliorate the effects of cancer cachexia, with resistance training being specially interesting given its potential to elicit an anti-inflammatory response and stimulate muscle protein synthesis (Leal et al., 2021). When only resistance training is used, it has been shown to be associated with clinical improvements in muscular function and body composition during and in the long term after cancer treatment (Strasser et al., 2013). Indeed, resistance training improved lower- and upper-body muscular strength in patients with breast cancer and potentially reduced breast cancer-related lymphoedema (Hasenoehrl et al., 2020). Furthermore, resistance training is associated with a lower risk of all-cause, cardiovascular disease, and cancer-specific mortality (Shailendra et al., 2022).

### Designing and Integrating Resistance Training into Multimodal Prehabilitation Programmes

The way resistance training programmes are configured determines their outcomes. As such, the following variables should be considered: exercise modality, determined by the technique, involved muscle mass, and type of muscle contraction (concentric, eccentric, or isometric); the order in which the exercises are performed; volume, estimated by multiplying the sets by the number of repetitions per set completed for each exercise (exercises × sets × repetitions); intensity, determined by the load and the movement velocity, where the load should be expressed in percentage of the one repetition maximum (1RM) defined by the amount of load (100%) that can be moved once but not twice in a consecutive movement for a given exercise (ACSM, 2017); inter-set rest intervals; and training frequency, determined by the number of workouts per week and recovery time between sessions.

Despite the benefits resistance training provides to patients with cancer, in our opinion, there is still a lack of consensus regarding the guidelines for designing resistance training programmes for patients with cancer undertaking prehabilitation. For instance, Carli et al. (2017) highlighted the need to precisely define the kind of physical activity incorporated into a planned and structured programme in contrast to generic recommendations of increasing physical activity before surgery. Additionally, these authors argue about the lack of specific guidelines to design endurance or resistance exercise programmes in the prehabilitation period. The ACSM states that exercise is safe for patients with cancer during and after treatment (ACSM, 2017). However, the designed programme requires individualised exercise modalities, intensities, and frequencies for each patient based on the kind of cancer and overall context (ACSM, 2017; Campbell et al., 2019). As such, it has been recommended that moderate-to-vigorous intensity physical activity should be performed at least 5 days per week for 30–60 minutes per day (ACSM, 2017). Aerobic exercise should be performed 2–5 times per week, resistance exercise 2–3 days a week, and flexibility exercise no less than 2 days a week and ideally every day (ACSM, 2017). Furthermore, in their latest consensus statement, the ACSM recommends that to address the health-related outcomes experienced due to cancer or cancer treatment, moderate-intensity aerobic exercise should be performed at least 3 times per week, for a minimum of 30 minutes and for a period of at least 8–12 weeks (Campbell et al., 2019) while integrating resistance (≥2 times per week, ≥2 sets of 8–15 repetitions per exercise, using relative loads of ≥60% of the estimated 1RM) to aerobic exercise seems to provide similar benefits (Campbell et al., 2019).

The latest exercise medicine in cancer management position statement by the Exercise and Sports Science Australia (Hayes et al., 2019) provides a thorough list of recommendations to design exercise programmes for patients with cancer based on the type of cancer, the treatment, or their side-effects. However, these guidelines do not detail in depth all variables influencing the design of strength training programmes. For instance, volume and intensity are described as low, moderate, or high without instructing further guidelines about how to choose the appropriate exercise intensity and training volume. Indeed, the recent publication by Champ et al. (2023) suggests fine-tuning the training variables to potentiate the physiological adaptations resulted from resistance training (i.e., improvements in balance, bone mineral density, hypertrophy). Champ et al. (2023) propose the use of at least 80%1RM for no more than 8 repetitions per set to promote increases in muscle mass and bone mineral density, while using multi-joint, free-weight exercises would have a positive impact on balance and proprioception. In this respect, free-weight multi-joint exercises loading the spine and hips (e.g., squat) would be especially important to reduce the risk of falls and improve the overall functional capacity of patients (Champ et al., 2023). To further clarify and help healthcare professionals design resistance training programmes for patients with cancer undertaking multimodal prehabilitation, we propose the consideration of the following aspects.

#### Exercise Selection

Sessions should involve 5 to 7 exercises. Priority should be given to multi-joint dynamic movements such as squats, leg presses, seated rows, and walking lunges over single-joint dynamic or isometric exercises (e.g., knee extension, calf raises, or arm curls) (Ratamess et al., 2009).

#### Workout Organization and Order of Exercise Execution

Start the workout with a 5- to 10-min warm-up, including dynamic stretching (e.g., lunge walking), light-intensity aerobics (e.g., brisk walking), and resistance multi-joint exercises using very light loads (ACSM, 2017). Thereafter, perform three to five multi-joint exercises (e.g., squats, lateral pull-downs, and leg presses), followed by two to three single-joint movements such as leg extensions and bicep curls (Ratamess et al., 2009). The session should end with a short 5–10-min cooldown involving gentle stretches and light-intensity cardiovascular activities (ACSM, 2017).

#### Workout Volume

It has been recommended the completion of 2–3 sets of a minimum of 8 to a maximum of 12 repetitions per exercise (Campbell et al., 2019; Ratamess et al., 2009). Performing 5 to 7 exercises configures a sensible approach, resulting in a total workout volume ranging from 80 to 315 repetitions.

#### Intensity

As previously mentioned, the intensity in resistance training is determined by the relative load (%1RM) and the velocity of execution. Although different %1RM can lead to muscle accretion and strength development (Ratamess et al., 2009), using higher %1RM can provide further physiological adaptations (e.g., increases in bone mineral density) (Champ et al., 2023). The last review by Champ et al. (2023) suggests that provided there is no contraindication to exercise with high to maximum loading ranges and patients can maintain an appropriate technique, exercises should be performed with at least 80% of 1RM.

In line with the suggestions of other researchers (Henkin et al., 2023) and current advice to control resistance training efforts in healthy adult individuals (Chapman et al., 2021; Robertson et al., 2003), we recommend using the OMNI Resistance Exercise Scale (OMNI-RES) to both determine the initial load and terminate the set by linking the recommended number of repetitions to the desired OMNI-RES score. As such, to perform a set of 8 repetitions using 80% of the estimated 1RM, an OMNI-RES score of 6–7 rated at the beginning of the set will be used to choose the training load and a final score of 8–9 (hard but not extremely hard) will indicate that enough effort has been achieved and the set should finish at that point.

#### Rest Period Between Sets of Exercises

The length of inter-set rest periods should be long enough to recover the neuromuscular capacity so that the consecutive sets can be performed at the same intensity with a similar perception of effort. This may also improve patients’ perception of exercise, supporting a more enjoyable training experience, avoiding excessive exhaustion, and likely promoting adherence (Lee et al., 2016). Overall, between 2 and 3 min have been recommended (Ratamess et al., 2009).

#### Training Frequency and Rest Between Workouts

A minimum frequency of two and ideally three sessions per week, performed on alternate days, is recommended (Ratamess et al., 2009). When both endurance and strength training modalities are combined within the same session, it is recommended to start with the resistance training routine to prevent the endurance-exercise-induced fatigue that can negatively impact force production (Kang & Ratamess, 2014).

#### Session Structure

A typical three weekly 60-min resistance training workout for patients with cancer involves the following three phases. A 10-min warm-up composed of stretching and light-intensity activities. The main phase should include two to three sets of 8–12 repetitions four to five lower and lower body multi-joint exercises (e.g., squats, chest press, lunges, and lateral pull-down), and two to three single-joint exercises (e.g., knee extension and biceps curl). A 2–3 minutes inter-set rest period is recommended. As previously described, the initial load should be selected based on the associated OMNI-RES score of 6–7 (estimated at the beginning of the set) with the expectation to reach the target final OMNI-RES score (8 or 9) within the recommended number of repetitions (8–12). Patients should prioritise a correct exercise technique while performing all repetitions with moderate to fast movement velocity during the concentric phase and slow, controlled movement during the eccentric phase. Finally, the cool-down phase (∼10 minutes) should include stretching and light intensity activities.

### Control of Training Progression

To ensure the training programme induces the expected physiological and mechanical outcomes for patients with cancer, a gradual increase of stress placed upon the body during exercise training is necessary (Ratamess et al., 2009). In unfit individuals, physiological adaptations to resistance training may occur in a short period (Lacio et al., 2021). Systematically increasing the demands placed upon the body is necessary for further improvement and may be accomplished through a thorough control of the level of effort experienced by the patients while exercising. As previously indicated, we suggest using the perceptual response to rate the level of effort to adjust the overload and number of repetitions performed for each singular exercise. As such, when patients complete the targeted number of repetitions (i.e., 8) rating an OMNI-RES score lower than the target (<8), they will be instructed to increase the number of repetitions toward a maximum of 12 per set while maintaining a final OMNI-RES score of 8–9. Thereafter, a minimum amount of load will be added as long as it is still possible to complete a minimum of 8 repetitions per set within the targeted OMNI-RES score expressed at the end of the set (i.e., 8–9) (Naclerio et al., 2015). This approach will permit patients and clinicians to follow a progressive effective training overload while maintaining the rate of perceived effort within the targeted training zone.

## Conclusions

The current paucity of research providing solid scientific evidence and limited resources precludes the UK’s National Health Service from integrating multimodal prehabilitation as part of the standard of care. Resistance training constitutes a crucial component in multimodal prehabilitation programmes. Clinicians and other health care professionals may consider the aforementioned recommendations to design and control patients’ progression during multimodal prehabilitation programmes.

## Futures Perspective and Final Recommendations

To allow for replicability and build a robust and consistent body of science, we invite researchers to thoroughly report their methodology related to the application of exercise programme intervention. Such clinical interventions are needed to ascertain the effects that multimodal prehabilitation may have on the clinical outcomes of surgical patients with cancer and, therefore, justify its integration within the National Health Service. Healthcare professionals should consider all the previously described training variables when implementing resistance training programmes for patients with cancer, whether as stand-alone interventions or as part of a multimodal programme (e.g., multimodal prehabilitation). To reach a consensus on designing resistance training for patients with cancer, future studies should incorporate standardised protocols, considering the above recommendations and reporting the observed effects on physical function, body composition and overall quality of life.
